# Leveraging deep learning and explainable AI for effective liver tumor classification from CT scan images

**DOI:** 10.3389/fonc.2026.1836325

**Published:** 2026-06-02

**Authors:** Meshal Alfarhood, Shatha Alotaibi, Aows Abuhaimed, Abdalrahman Alalwan

**Affiliations:** Department of Computer Science, College of Computer and Information Sciences, King Saud University, Riyadh, Saudi Arabia

**Keywords:** classification, deep learning, explainable artificial intelligence, liver cancer, liver tumors

## Abstract

**Introduction:**

Cancer remains one of the leading causes of mortality worldwide, with 19.3 million new cases and 10 million deaths reported in 2020. According to the World Cancer Research Fund International (WCRF), liver cancer ranks as the fifth most prevalent cancer in men and the ninth in women. Despite available interventions, liver cancer is often diagnosed at advanced stages due to its subtle progression and the complexity of distinguishing hepatic malignancies from surrounding tissues in CT scans. Conventional diagnostic practices, such as biopsy, are invasive, time-consuming, and mentally exhausting for patients, while manual interpretation of CT images is labor-intensive and requires expert radiologists. These challenges highlight the urgent need for automated, accurate, and explainable diagnostic tools.

**Methods:**

In this work, we propose a comprehensive deep learning framework for non-invasive liver tumor classification with integrated explainability. We evaluated and fine-tuned several state-of-the-art supervised models, including ResNet50-v2, EfficientNetV2, Inception-v3, and Vision Transformer ViT-16, combined with tailored pre-processing and augmentation strategies.

**Results and discussion:**

The EfficientNetV2 model achieved 96.97% accuracy, demonstrating competitive performance with existing literature. Beyond high accuracy, the framework integrates explainable AI methods to enhance interpretability and clinical trust, bridging a key gap in current AI-driven liver cancer research.

## 1 Introduction

Liver cancer is one of the leading causes of cancer-related mortality. In 2020, over 900,000 new instances of liver cancer were documented globally. Final diagnosis traditionally relies on biopsy, which is painful and carries procedural risk, while non-invasive CT analysis depends on radiologists expert doing manual readings, which show inter-observer variability and reported AUC values as low as 0.81 Blachar et al. (1). With these constraints about the cost of delay diagnostics, limited availability of the radiologist expert, and the burden of invasive confirmation shows a real need for an accurate, automated and clinically interpretable diagnostic tools.

Recent advancements in deep learning have markedly enhanced medical picture analysis. Ensemble-based networks have exhibited strong efficacy in tumor classification tasks by amalgamating various architectures to enhance generalization Hekmat et al. (2). Multimodal deep learning frameworks, as demonstrated by MeD-3D, utilize CT, MRI, histology, and genetic data to enhance recurrence prediction, highlighting the efficacy of cross-modality integration Maqsood and Khan (3). Attention-based transformer models have demonstrated efficacy in extracting intricate features across many scales, enhancing the accuracy of tumor detection and classification Khan et al. (4). Although these methodologies yield robust outcomes, they are predominantly constrained by dependence on manually produced features or limited datasets, and few include explainable AI (XAI) techniques that enhance transparency and foster therapeutic trust.

While the model architectures including (ResNet, EfficientNet, Inception, ViT) and explainable artificial intelligence (XAI) methods including (SHAP, Grad-CAM, Saliency) used in this work have been applied in similar problems before, our contribution lies in their integration within a framework dedicated to classifying liver tumors from CT scans along with integrating XAI methodologies to overcome existing gaps, complementing prior efforts that have primarily addressed segmentation or classification without interpretability. The main contributions of this proposed work are as follows:

1. Development of a three-step automated liver diagnosis system, including preprocessing, classification, and explainable classification.
2. Experimental evaluation of multiple state-of-the-art architectures (ResNet50-v2, EfficientNetV2, Inception-v3, and ViT-16) on a collected CT liver dataset.
3. Integration of explanation models (SHAP, Grad-CAM, and Saliency Maps) to enhance interpretability and build clinical trust in predictions.

The remainder of this paper is organized as follows. Section 2 reviews the related literature, highlighting key studies and methodologies relevant to our work. Section 3 describes the proposed methodology, detailing the complete pipeline from data acquisition and preprocessing to CT image classification and model explainability. Section 4 outlines the experimental setup used to conduct this study. Section 5 presents and analyzes the results, discussing the main findings and their limitations. Finally, Section 6 concludes the paper by summarizing the primary contributions and suggesting directions for future research.

## 2 Related work

This section presents a focused review of prior academic and scientific work related to liver tumor classification, with particular emphasis on methods based on CT image analysis. It also surveys recent advances in interpretable and explainable AI models for medical imaging, highlighting approaches that aim to improve model transparency, clinical trust, and decision support.

### 2.1 CT image classification

Recent research in biomedical imaging has explored a wide range of classification approaches for tumor detection in different organs, including the brain, lung, colon, breast, and liver. While some systems apply the same models across multiple organs, others adapt architectures specifically to each task. In addition to liver tumor classification, studies on breast, lung, and brain cancer provide useful information on the strengths and limitations of current methods.

Hussain et al. Hussain et al. (5) emphasized the importance of developing fully automated approaches for the detection of liver tumors using CT images. Their study implemented several machine learning (ML) classifiers in regions of interest (ROI) of varying dimensions (11×11 to 21×21). The smaller ROIs produced suboptimal accuracy, but performance improved significantly at larger ROIs, reaching 97.48% accuracy with 17×17 dimensions. Although promising, this ROI-based approach is limited in scalability when applied to large and complex datasets.

Hekmat et al. Hekmat et al. (2) proposed an ensemble deep learning framework optimized for medical image classification. Their model combined multiple CNN-based architectures to improve robustness and predictive performance, achieving an accuracy of 98.7% on benchmark tumor datasets, outperforming individual CNN models. However, the study did not incorporate explainability, which limits its applicability in clinical workflows where interpretability is essential.

Naeem et al. Naeem et al. (6) proposed a hybrid-feature dataset combining histogram, wavelet, co-occurrence, and run-length characteristics to evaluate several ML classifiers, including a Multilayer Perceptron (MLP). Their model achieved 95.78% accuracy on MRI data and 97.44% on CT data. When MRI and CT features were fused, the accuracy increased to 99%. While the results are strong, the approach relies heavily on handcrafted features, which may limit adaptability to new or diverse datasets.

Maqsood et al. Maqsood and Khan (3) introduced MeD-3D, a multimodal deep learning framework for recurrence prediction in clear cell renal carcinoma. By integrating CT, MRI, histopathology, genomic, and clinical data, their framework achieved an overall accuracy of 95.6%, significantly higher than unimodal models. While effective, the system emphasizes multimodal integration rather than explainable classification, leaving interpretability as an open challenge.

Khan et al. Khan et al. (4) developed an attention-based transformer model tailored for tumor detection and classification in medical imaging. Leveraging multi-scale feature extraction, their approach reported an accuracy of 97.2%, outperforming standard CNN baselines. Despite this strong performance, the model operates as a black box, without incorporating explainability techniques necessary for clinical trust.

Balasubramanian et al. Balasubramanian et al. (7) developed a three-stage pipeline involving preprocessing, liver segmentation using Mask R-CNN, and classification with an Enhanced Swin Transformer Network and Adversarial Propagation to reduce overfitting. Their method achieved 94.32% accuracy on abdominal CT images. However, the study did not incorporate model interpretability, which is critical for clinical application.

To summarize the architectural distinctions between CNNs and attention-based models described earlier, **Table 1** compares the key mechanisms, strengths, and limitations of these models. Expanding on these architectural contrasts, additional research, such as that of Devi and Seenivasagam Devi and Seenivasagam (8), has investigated computer-aided diagnostic systems that incorporate segmentation and classification methodologies.

**Table 1:** Architectural comparison of CNN and attention-based models relevant to our work.

Model	Type	Mechanism	Strengths	Limitations
ResNet50-V2	CNN	Residual connections enabling deep CNN training	Stable optimization; strong baseline performance	Limited global context; long-range dependencies
EfficientNetV2-S	CNN	Compound scaling of depth, width, resolution; fused MBConv blocks	Excellent accuracy–efficiency trade-off; lightweight	Still CNN-local; limited ability to model long-range context
Inception-v3	CNN	Parallel multiscale convolution (Inception modules)	Captures multi-scale features effectively	Higher compute; limited global context modeling
ViT-16	Transformer	Patch embedding + self-attention across tokens	Models long-range dependencies; flexible representation	Data-hungry; can overfit on small medical datasets
Swin Transformer	Transformer	Hierarchical transformer with shifted windows	Scales to larger images; captures local & global context	More complex; higher training cost

Devi and Seenivasagam Devi and Seenivasagam (8) introduced a computer-aided diagnosis (CAD) system combining automated liver segmentation, lesion identification, and a novel contrast-based feature difference technique. Using these features, an SVM classifier distinguished malignant and benign lesions with 98.6% accuracy. Although accurate, the reliance on handcrafted features limits transparency and reproducibility. Similarly, Rela et al. Rela et al. (9) applied a fast Fuzzy C-Means (FCM) clustering algorithm for liver segmentation in CT images. While their method was efficient and accurate on a small dataset of 20 images, it remains limited by dataset size and lack of external validation.

Addressing the limitations of purely 2D or 3D feature extraction, Meng et al. Meng et al. (10) proposed a hybrid framework that integrates both 2D and 3D features with a densely connected UNet (DCUNet) and an attention mechanism to capture multi-scale tumor features. On the LiTS dataset, they reported Dice coefficients of 0.967 for liver segmentation and 0.725 for tumor segmentation. Although effective in segmentation, the study placed less emphasis on tumor classification and interpretability. Kutlu and Avcı Kutlu and Avcı (11) developed a CNN–DWT–LSTM hybrid model that combined convolutional networks, wavelet transforms, and recurrent layers. Their model achieved 99.1% accuracy for liver tumor classification and 98.6% for brain tumors, outperforming traditional classifiers such as SVM and k-NN. Despite its performance, the study lacked clinical explainability and external dataset validation.

Ensemble and deep learning methods have also been applied in other cancer domains. Assiri et al. Assiri et al. (12) proposed an ensemble classification approach with majority voting, achieving success rates of up to 99.42%. Rahman et al. Rahman et al. (13) adapted pre-trained CNNs to detect breast masses in mammography images, reporting accuracies of 79.6% with an InceptionV3-like model and 85.7% with a ResNet50-like model. In lung cancer, Asuntha and Srinivasan Asuntha and Srinivasan (14) combined deep learning with feature extraction and optimization techniques to detect malignant nodules, achieving 99.13% accuracy. These studies highlight the broad utility of AI in cancer imaging but also reveal recurring issues: dependence on handcrafted features, lack of interpretability, and limited reproducibility.

Summary and Research Gap: The literature demonstrates strong progress in tumor classification and segmentation using ML and DL methods. However, three consistent gaps remain:

1. Numerous models demonstrate elevated accuracy; however, they rely on manually constructed characteristics or limited datasets, which constrain their generalizability.
2. Limited methodologies systematically evaluate contemporary CNNs and Transformers for the categorization of liver tumors.
3. The majority of studies prioritize segmentation or detection, and a limited number focus on holistic classification frameworks that include accuracy and robustness.

### 2.2 Interpretable AI models for medical imaging

Specific AI systems can be highly complex and opaque, limiting immediate understanding of their decision-making processes. In the medical field, this lack of transparency is critical, as physicians and patients can only fully trust AI systems if the rationale behind predictions is communicated clearly. Such transparency also allows errors and biases to be detected. Explainable AI (XAI) has therefore emerged as a growing area of research, aiming to develop methods that make AI systems more interpretable and clinically trustworthy. This subsection reviews several works that have incorporated interpretable AI models in medical imaging.

Rucco et al. Rucco et al. (15) introduced topological features as a novel set of radiomic descriptors for glioblastoma multiforme (GBM) analysis using FLAIR MRI. Their contributions included: (i) applying topological data analysis (TDA) to study GBM progression, (ii) proposing a new entropy measure, Generator Entropy, analogous to Shannon’s entropy, and (iii) integrating topological and textural features to train interpretable ML models. Validated on FLAIR MRI data, their approach achieved 97% classification accuracy. While effective, this method relies on specialized topological descriptors, which may limit broader applicability across different imaging modalities.

Metta et al. Metta et al. (16) focused on improving user trust in AI systems for skin lesion diagnosis by customizing an XAI method to generate case-specific explanations. Their approach employed synthetic images as examples and counterexamples, helping practitioners identify the features most influential in classification outcomes. Although the study demonstrated that saliency maps from their ABELE explainer outperformed those of LIME and LORE, the authors also highlighted persistent skepticism among older clinicians and reduced confidence when incorrect AI predictions were observed—emphasizing the need for clinically reliable explainability methods.

In a related study, Hassan et al. Hassan et al. (17) employed an enhanced DenseNet201 for feature extraction, integrating Local Interpretable Model-agnostic Explanations (LIME) to provide transparency in COVID-19 diagnosis from chest images. Their framework improved both accuracy and interpretability, allowing physicians to better understand the model’s reasoning. Similarly, Schutte et al. Schutte et al. (18) introduced an interpretability technique based on StyleGAN, which generates synthetic image variations to illustrate how predictions change with input alterations. Their approach provided deeper insights than conventional GradCAM heatmaps and was successfully applied to histology and radiology images.

Pitroda et al. Pitroda et al. (19) developed a CNN framework for classifying lung diseases, including COVID-19, from chest X-rays. To address the black-box nature of CNNs, they incorporated Layer-wise Relevance Propagation (LRP), offering clinicians a quantitative understanding of the model’s predictions. Wu et al. Wu et al. (20) proposed a Joint Classification and Segmentation (JCS) model for real-time COVID-19 CT diagnosis, producing both classification outputs and lesion segmentation maps. This dual output provided radiologists with interpretable, clinically relevant information, improving diagnostic confidence.

**Table 2** presents a summary of previous studies, emphasizing their fundamental mechanisms, documented performances, and principal constraints. Notwithstanding their strong results, several models depended on manually generated features, exhibited a lack of interpretability, or were limited by the dataset’s size and variety. The persistent constraints underscore the need for an integrated framework that combines contemporary deep learning architectures with interpretability, while ensuring scalability and clinical reliability. The ongoing challenges motivate our proposed methodology, which we delineate in the subsequent section.

**Table 2:** Comparative analysis of recent studies on tumor classification and their limitations compared to our proposed framework.

Study	Accuracy	Limitation	How our work addresses it
Hussain (5)	97.48% (CT, ROI 17×17)	ROI-based; limited scalability for complex datasets	End-to-end classification on full CT images, eliminating ROI dependency
Hekmat (2)	98.7%	Ensemble CNNs but no explainability, limiting clinical trust	Integration of XAI (SHAP, Grad-CAM, Saliency) to provide interpretable outputs
Naeem (6)	95.78% (MRI), 97.44% (CT), 99% (fused)	Substantial reliance on handcrafted features; limited adaptability	Automated feature extraction via DL architectures (CNNs + Transformer)
Maqsood and Khan (3)	95.6%	Multimodal integration; lacks explainable classification	Focus on single-modality CT with interpretable classification framework
Khan et al. (4)	97.2%	Attention-based Transformer; strong accuracy but black-box	Uses ViT alongside CNNs with XAI to enhance interpretability
Balasubramanian et al. (7)	94.32%	Segmentation + classification, but no interpretability	Adds explainable models for classification (XAI integrated)
Devi and Seenivasagam (8)	98.6%	Handcrafted contrast-based features; limited reproducibility	DL pipeline with preprocessing + augmentation for generalizability
Meng et al. (10)	0.967 (liver), 0.725 (tumor)	Focused on segmentation, less on classification/XAI	Direct tumor classification with interpretable AI outputs
Kutlu and Avcı (11)	99.1% (liver), 98.6% (brain)	Hybrid CNN–DWT–LSTM; lacks explainability, limited validation	Pure DL models benchmarked systematically with XAI

Summary and Research Gap: The analyzed studies demonstrate the growing importance of explainable AI (XAI) in medical imaging. Methods, including saliency maps, LIME, and gradient-based visualizations, have enhanced transparency in areas such as brain, lung, and skin tumors. However, their use in liver categorization is limited, and few studies integrate sophisticated designs with explainability within a comprehensive diagnostic framework.

## 3 Materials and methods

This work proposed a system that consists of two parts: classification using liver CT scans and explainable models that look inside the black box of classification decisions. Several classification architectures were used to classify liver tumor images as normal, benign, and malignant. These images were collected from Kaggle Liv (21) and the Radiopaedia Rad (22) websites. Data pre-processing and data augmentation approaches were employed to reduce noise, improve image pixels’ clarity for better differentiation, and address the issues of small datasets before starting training. This section explains these proposed approaches.

### 3.1 Overall workflow

**Figure 1:**
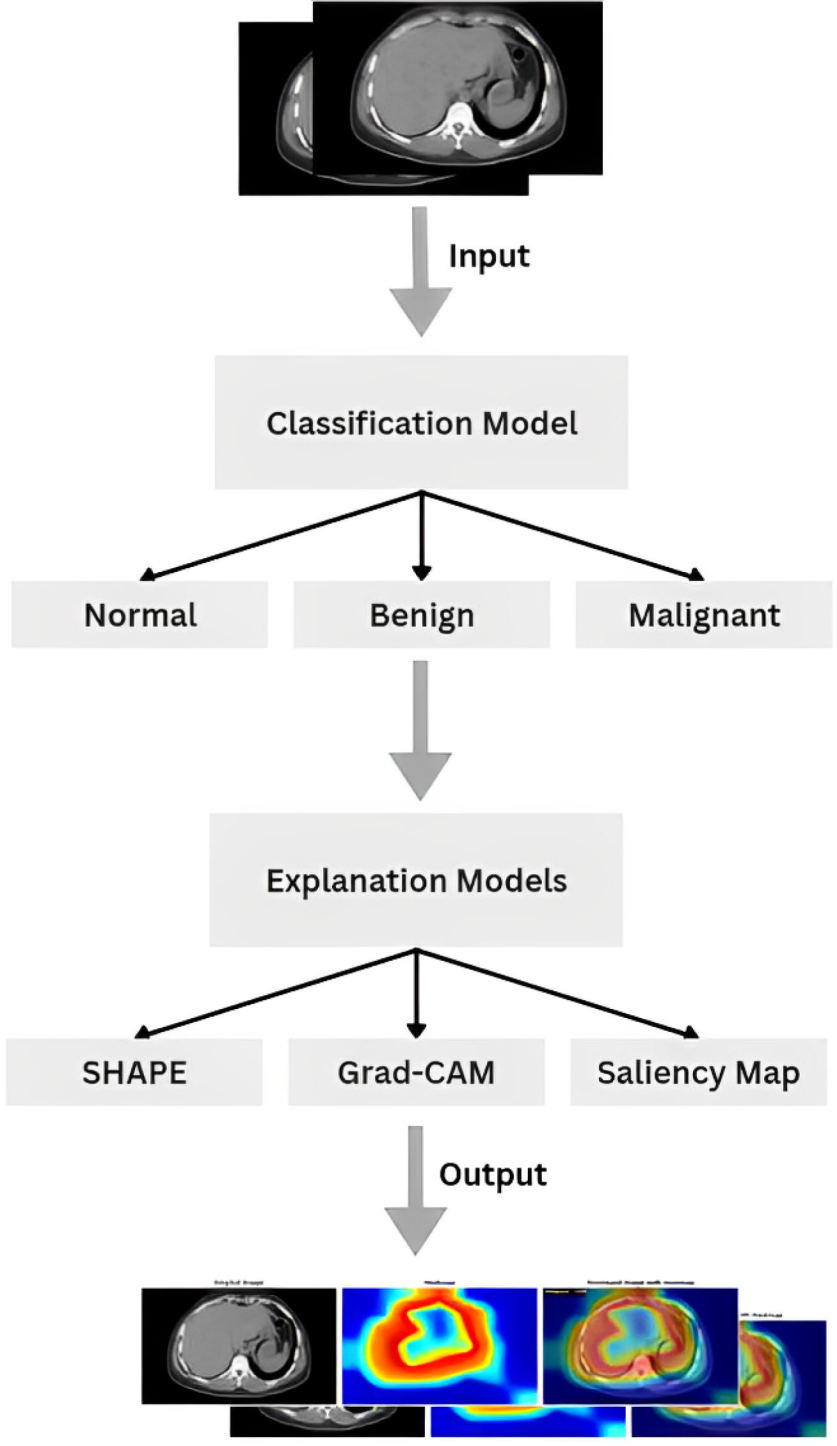
Overall workflow of the proposed liver tumor classification framework. It illustrates the two major components of the system: classification of CT liver images and the integration of explainable AI methods for interpretability.

This research proposes a system that could help radiologists and specialists in their work. Moreover, the classification approach was followed by explanation models to understand the reasons behind these decisions. **Figure 1** illustrates the overall workflow. Our ultimate goal is to provide complete information about a given CT scan, its class, and the results of the explainable model for the classification. For that, the following subsections address the methodology of classification and the explainable models in complete form. In practice, the system is intended to be used as a decision support tool for the radiologists rather than a replacement. The radiologist feeds the CT image to the model, receives the predicted classification whether normal, benign, or malignant along with XAI visualizations against the suspected lesion location to confirm or escalate the case.

### 3.2 Liver CT images classification

Recent advancements in artificial intelligence have created very efficient algorithms for many medical diagnostic purposes. One problem often encountered is the categorization of liver tumors Kiani et al. (23). AI algorithms have demonstrated great accuracy in specific tumor classification tasks Alom et al. (24). The training of the liver tumor classification models in this work was done using abdominal CT images constituting our dataset, we call “CT Liver Mix Data.” Then, the test split of this dataset was used to evaluate the classification robustness. This section provides an insight into the classification process, explainable classification, and all the steps taken on the dataset.

#### 3.2.1 Classification workflow

**Figure 2:**
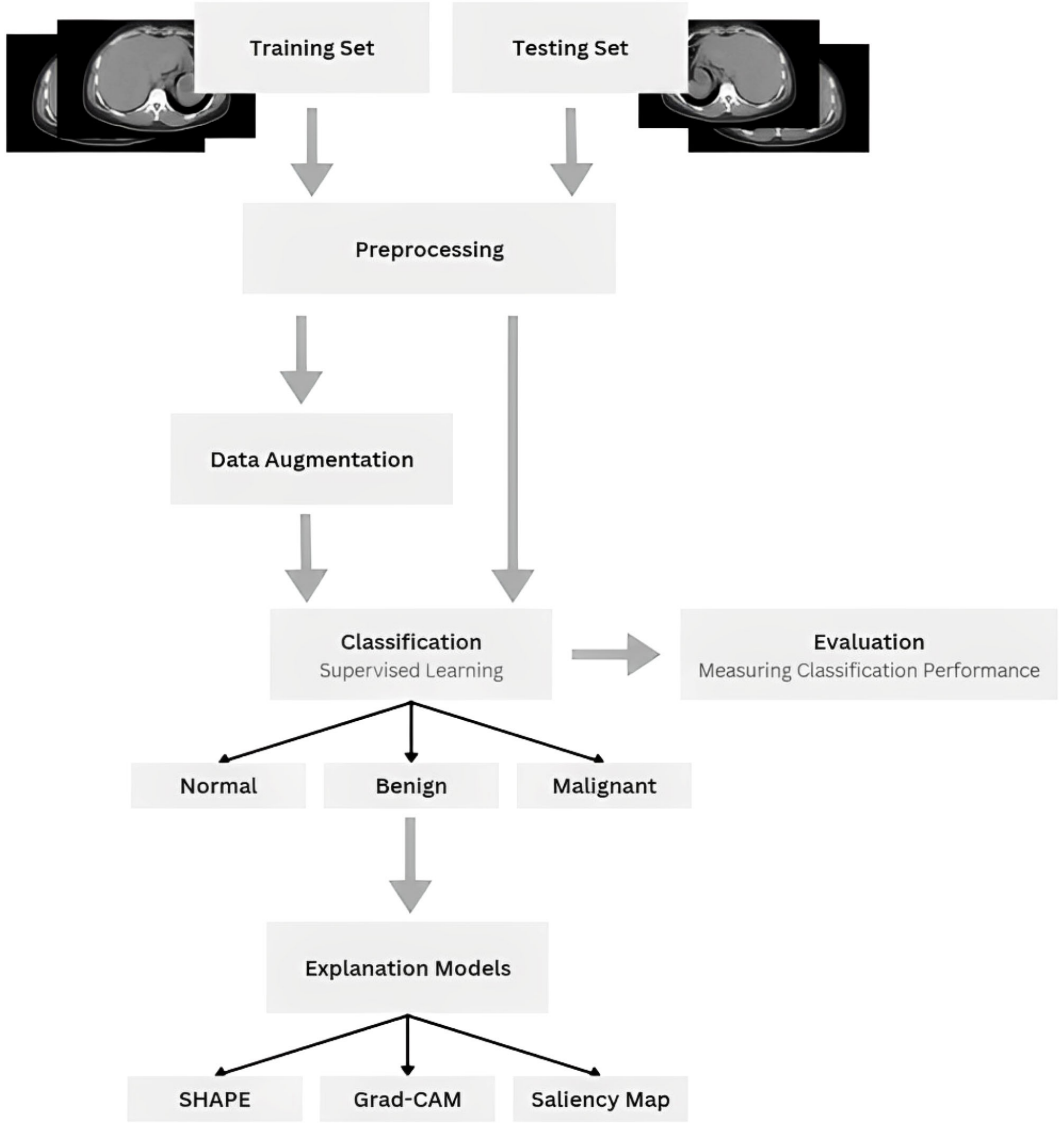
Classification methodology workflow. Step-by-step pipeline for liver tumor classification, including dataset preparation, preprocessing, augmentation, model training, evaluation, and explainability.

The classification approach aims to classify liver lesions in CT scans as normal, benign, and malignant. **Figure 2** presents an overview of our proposed method, which consists of the following steps: Dataset, Data Pre-processing, Data Augmentation, Classification, Evaluation, and Explanation Models.

Initially, the CT images in our dataset were split into training and testing sets. After that, all the datasets were pre-processed by performing two techniques: denoising using a bilateral filter and histogram equalization to extend the processed CT image pixels nonlinearly. The following technique was data augmentation, performed on a training set only. Next, several deep learning architectures were used to obtain classification and extract the features. The last stage of classification was the evaluation using the test set to measure the performance of the classification model. After the classification, we initiated our explanation models to comprehend the justifications for these predictions.

#### 3.2.2 Dataset

The dataset used here is mixed data from two datasets for the classification models. The first is the Liver Tumor Classification dataset from Kaggle Liv (21), and the second is from the Radiopaedia website Rad (22). Both were used as one dataset, “CT Liver Mix Data,” to train the classification model; the number of images in this dataset is illustrated in **Table 3**. The dataset is balanced across the three classes (Normal: 104 [31.8%], Benign: 120 [36.7%], Malignant: 103 [31.5%]), with a maximum-to-minimum gap of only ∼5%. Therefore, no data imbalance techniques were used. Instead, random shuffling was applied to ensure the model observers a mix of samples during training. The images in both datasets have been visually examined to rule out the existence of personal identifiers, and all data have been anonymized Bilic et al. (25).

**Table 3:** Liver tumor classification dataset distribution.

Class	Training set	Validation set	Testing set
Normal	63	20	21
Benign	73	23	24
Malignant	62	20	21

#### 3.2.3 Data preprocessing

The proposed system involves several combinations of pre-processing steps, including resizing the image, histogram equalization, and noise reduction techniques as needed to improve the performance of the proposed method.

##### 3.2.3.1 Histogram equalization

Histogram equalization approaches Pizer et al. (26) have been extensively utilized in image processing to enhance the contrast and visibility of structures. The methods use the image’s overall intensity distribution for feature enhancement, as defined by the normalized cumulative histogram. The technique of the CLAHE Singh et al. (27), used in this work, separates the image into many tiles. The tile size is determined according to the grid-size option. The grid-size option is a tuple (rows, columns) specifying the number of rows and columns used to split the image for the CLAHE application. CLAHE divides the image into smaller tiles and applies histogram equalization locally to each tile to prevent noise over-amplification in uniform areas and enhance local contrast. For the experiment Singh et al. (27), grid-size 8x8 was used.

##### 3.2.3.2 Noise reduction

The bilateral filter was suggested, described in Tomasi and Manduchi (28), as a technique for noise reduction. The bilateral filter effectively reduced noise in CT images by smoothing image details while maintaining sharp edges using a nonlinear blend of neighboring pixel values. An intricate filter kernel that combines spatial and intensity range elements accomplishes this Wagner et al. (29). The approach is noniterative, local, and straightforward. The process mixes gray levels or colors by considering their geometric proximity and morphometric similarity, giving preference to closer values in both domains and ranges. Unlike filters that work on individual color channels of an image, a filter designed for the CIE-Lab color space can enhance perceptual symmetry and smooth colors and maintain edges in a manner aligned with human perception. Bilateral filtering differs from ordinary filtering by creating phantom colors in the images and removing phantom colors in the original image Tomasi and Manduchi (28).

#### 3.2.4 Data augmentation

The potential for overfitting exists due to the datasets’ restricted size. To address this; we applied on-the-fly data augmentation during training to introduce random variations such as rotations, translations, scaling, and flips. This research used random rotations (up to ±20°), horizontal and vertical shifts (up to 6% of the image dimensions), and random horizontal and vertical flips. These techniques ensures the model observes new variations of the same images in each training epoch without altering overall dataset size.

#### 3.2.5 Classification models

Deep learning methodologies can effectively tackle the obstacles inherent in the field of medical image processing Saha Roy et al. (30). Hence, the following architectures were experimented with and evaluated to achieve the best performance: Residual Network (ResNet50-V2) He et al. (31), EfficientNetV2-S Tan and Le (32), Inception-V3 Szegedy et al. (33), and Vision Transformer Dosovitskiy et al. (34). All of these architectures have been subjected to transfer learning methodology.

##### 3.2.5.1 Residual Network (ResNet50-V2)

The ResNet50 model He et al. (35) is a convolutional neural network trained on the extensive ImageNet dataset, designed explicitly for object recognition tasks. In recap, the fundamental principle underlying ResNet involves incorporating an identity shortcut connection, which allows for bypassing one or several layers. The employed methodology utilizes skip connections, which establish connections between two to three layers of ReLU and batch normalization inside the architectural framework. This feature enables the network to effectively align the residual mapping instead of facilitating the acquisition of the underlying mapping He et al. (35)Islam et al. (36). The main idea underlying ResNets is to train the residual function F concerning *h*(*x_l_*), using an identity mapping *h*(*x_l_*) = *x_l_*; this is accomplished by incorporating an identity skip connection (shortcut). However, this work He et al. (31) investigates deep residual networks by building a direct conduit for conveying information within a residual unit and throughout the entire network. Their derivations show that when both *h*(*x_l_*) and *f*(*y_l_*) are identity mappings, the signal can be sent directly from one unit to any other units in both the forward and backward passes. The results indicate that training becomes more tractable when the architecture closely matches the conditions above. Moreover, a pre-trained ResNet50-V2 Res (37) was used in the experiment, and the last layers were fine-tuned to fit the specific task requirements of this work, using the architecture proposed in Chakraborty et al. (38), and we added at the head of the classifier, four layers: Global average pooling 2d layer with 2048 nodes, Dense layer (Fully Connected) with 1024 nodes, and ReLU (Equation 2) as its activation function, Batch Normalization layer with 1024 nodes, and dense layer (Fully Connected) with three nodes and SoftMax (Equation 1) as activation function.

(1) $$\sigma \left(y_{i}\right)=\left(\frac{e^{y_{i}}}{\sum_{j=1}^{n}e^{y_{j}}}\right)$$

(2) ReLU(x)=max (0,x)

The ResNet architecture introduces residual learning by reformulating the layers (Equation 3) as:

(3) $$y_{l}=F\left(x_{l},W_{l}\right)+x_{l},$$

where *x_l_*and *y_l_*are the input and output of the *l*-th layer, and *F* is the residual mapping He et al. (35).

##### 3.2.5.2 EfficientNetV2-S

The EfficientNet Tan and Le (39) architecture is a deep learning model that has gained significant attention and popularity due to its superior performance in specific computer vision tasks. It is widely recognized that enhancing the performance of Convolutional Neural Networks (CNNs) requires adjusting several parameters, such as depth, width, or resolution. EfficientNetV2-S differs from the EfficientNet backbone in several notable ways. EfficientNetV2-S extensively utilizes MBConv and the recently announced fused MBConv in its early layers. Moreover, EfficientNetV2-S prioritizes a decreased expansion ratio in MBConv, as smaller ratios typically lead to less memory access overhead. EfficientNetV2-S emphasizes using smaller 3x3 kernel sizes and compensates for the reduced receptive field created by these smaller kernels by adding extra layers. EfficientNetV2-S removes the last stride-1 operation employed in the original EfficientNet, most likely due to its substantial parameter size and intricacy in memory retrieval Tan and Le (32).

In this work, pre-trained EfficientNetV2-S Eff (40) was utilized and fine-tuned as performed in ResNet50-V2 architecture in the previous subsection. Still, in EfficientNetV2-S, we did not add a dense layer after the Global Average Pooling (GAP) layer because the model performed better without it. We also added a dropout layer to reduce the overfitting in the model. The architecture of this model, as in Song (41), but with the modification on the last layers.

EfficientNet employs compound scaling to balance depth (*d*), width (*w*), and resolution (*r*), (Equation 4) defined as:

(4) $$d=\alpha^{\phi },\;w=\beta^{\phi },\;r=\gamma^{\phi },$$

subject to *α* · *β*^2^ · *γ*^2^ ≈ 2, where *ϕ* is the scaling coefficient (32) Tan and Le (39).

##### 3.2.5.3 Inception-V3

The Inception architecture was initially developed as a case study to evaluate the potential output of an advanced algorithm for constructing network topologies. This algorithm aims to approximate a sparse structure suggested by Arora et al. (42) Szegedy et al. (43) for vision networks while utilizing dense and readily accessible components to cover the expected results. The intricate nature of the Inception architecture in Szegedy et al. (43) renders modifying the network more complex. To fully regain the computing advantages, it is necessary to scale up the design and provide a clear and comprehensive explanation of the influential variables. Under those circumstances, the straightforward modification of doubling the sizes of all filter banks results in a fourfold rise in both computational expense and parameter count. This could be impractical or unacceptable in numerous real-world situations, mainly if minimal benefits exist. The modification of Inception-V3 by utilizing fewer parameters and implementing additional regularization techniques, such as batch normalization, supplemental classifiers, and label smoothing, makes it possible to train high-quality neural networks even with relatively small training dataset. The Inception-V3 pre-trained model we used in this work was introduced in a previous publication by Szegedy et al. Szegedy et al. (33). This model consists of more than 20 million parameters and has been trained by one of the leading hardware specialists in the business. The model consists of symmetrical and asymmetrical construction pieces, including convolutional, average, max pooling, concats, dropouts, and fully connected layers. Furthermore, batch normalization is frequently utilized and implemented on the input of the activation layer in this model. The classification process is executed with the Softmax algorithm Ali et al. (44).

As with the previous models in this section illustrated, pre-trained Inception-V3 Inc (45) was used, and the head of this classifier has four layers to adapt to the given task as the same layers were added for ResNet50-V2. The architecture of this model is the same architecture in Ali et al. (44) but with the modification in the last layers.

The Inception-v3 architecture reduces computational complexity by factorizing convolutions (Equation 5), e.g., an *n* × *n* convolution can be approximated as:

(5) $$f\left(x\right)=W_{1}*\left(W_{2}*x\right),$$

where *W*_1_ and *W*_2_ are smaller convolutional kernels such as 1 × *n* and *n* × 1 Szegedy et al. (33).

##### 3.2.5.4 Vision Transformer ViT-16

Vision Transformer ViT-16 architecture Dosovitskiy et al. (34) has investigated the direct implementation of Transformers for image recognition. Unlike previous efforts that utilized self-attention in computer vision, the developers of this suggested system did not incorporate image-specific inductive biases into the architecture. The only exception was the initial patch extraction process. They analyze an image by breaking it down into patches and then apply a standard Transformer encoder similar to the one used in Natural Language Processing (NLP) Vaswani et al. (46). This straightforward yet adaptable approach is efficient with pre-training on extensive datasets. Vision Transformer achieves state-of-the-art performance on numerous image classification datasets and is cost-effective for pre-training.

Due to the small size of the dataset, pre-trained ViT-16 vit (47), which is very commonly used for limited datasets, was used. For that, freezing the first layers was necessary, and the head of the classifier was fine-tuned, using the same architecture as in this work Dosovitskiy et al. (34) but with our added layers in the head of the classifier.

The Vision Transformer (ViT) divides an image $x\in {\mathbb{R}}^{H\times W\times C}$ into a sequence of flattened 2D patches $x_{p}\in {\mathbb{R}}^{N\times \left(P^{2}\cdot C\right)}$, where $N=\frac{HW}{P^{2}}$ is the number of patches and *P* is the patch size. Each patch is linearly projected into an embedding (Equation 6):

(6) $$z_{0}=\left[x_{p}^{1}E;x_{p}^{2}E;\ldots ;x_{p}^{N}E\right]+E_{pos},$$

where *E* is the learnable projection matrix and *E_pos_*is the positional encoding. Dosovitskiy et al. (34) Then, self-attention (Equation 7) is applied inside the Transformer encoder:

(7) $$\text{Attention}\left(Q,K,V\right)=\text{softmax}\left(\frac{QK^{T}}{\sqrt{d_{k}}}\right)V,$$

where *Q*, *K*, and *V* are the query, key, and value matrices.

### 3.3 Explanation models

In recent years, there has been significant growth in research in machine learning Nguyen et al. (48). Multiple new regions have been created, while certain previously established areas have experienced a surge in activity. Despite being widely adopted, machine learning models predominantly exist as opaque entities. Comprehending the decisions and rationales behind the predictions made by models is crucial when evaluating the level of trustworthiness. Explainable Models are designed to explain the logic and rationale behind the decisions and predictions made by a system. Researchers from many disciplines focus on different objectives and distinct subjects in the study of Explainable Models, promoting interdisciplinary viewpoints on the aims of intelligibility and transparency Nguyen et al. (48). This subsection overviews the most used explanation methods in the image classification problem, including SHAP, GradCAM, and Saliency Map.

#### 3.3.1 SHapley Additive exPlanations

(SHAP) aim to explain model predictions by quantifying the contribution of each feature to the forecast Nguyen et al. (48)Lundberg and Lee (49). To achieve this objective, SHAP utilizes Shapley Values derived from game theory. Explaining the concept of Shapley Value is necessary to enhance comprehension of the investigated method. It is a technique in which game players are rewarded based on their overall contribution to the total benefit. The concept has been conveyed to SHAP to evaluate the features that substantially impact the prediction made by the final model. A coalition vector assigns a binary value of 0 or 1 to each characteristic, indicating its presence or absence in the coalition. The vectors are transformed into the feature space using the image function, which assigns gray super-pixels to vectors with a value of 0.

#### 3.3.2 Gradient-weighted class activation mapping

This approach produces visual explanations for decisions made by various Convolutional Neural Network (CNN)-based models, improving their transparency Selvaraju et al. (50). The Gradient-weighted Class Activation Mapping (Grad-CAM) strategy leverages the gradients of a specific target notion, such as the logits for ‘dog’ or a caption, which flow into the final convolutional layer. This procedure produces a rudimentary localization map that emphasizes the essential areas in the image that are significant for anticipating the concept. Unlike previous techniques, GradCAM applies to different families of CNN models, including those that contain fully connected layers, such as VGG. Grad-CAM combines current detailed visualizations to produce a high-resolution representation tailored to a particular class.

#### 3.3.3 Saliency map

Saliency maps are widely used to elucidate the inner workings of convolutional neural networks Amorim et al. (51). They demonstrate the significance of each pixel in the input image for the overall prediction of a CNN. The hue or magnitude of every pixel in the saliency map corresponds to the significance that the corresponding pixel in the input image has on the classification process. It has been discovered within the subject of oncology that most interpretability techniques used can be classified as belonging to the saliency map category Amorim et al. (51, 52).

## 4 Experimental settings

This section describes the experimental tools used to conduct and assess this research. The materials to be used to evaluate the proposed models are explained in the following sections.

### 4.1 Designing experiments

The utilized resources in this work are the following:

- Hardware: Since the proposed system requires high memory and GPU capacity, we used a local machine equipped with an NVIDIA RTX 4080 Super GPU. This setup provided the necessary computational power for our tasks.
- Software: The frameworks we employed during the experiment were PyTorch, TensorFlow, Monai, and the following Python libraries: SKlearn, Pandas, and NumPy.

Several pre-processing steps, such as resizing the image, histogram equalization, and noise reduction techniques, were applied to the collected dataset. For that, these three dependencies were needed:

- OS: The Python OS module offers routines that facilitate operating system interactions. Pyt (53).
- CV2: OpenCV is a collection of Python interfaces created to address computer vision challenges Pyt (53).
- NumPy: Numerical Python compatible array library Pyt (53).

**Figure 3:**
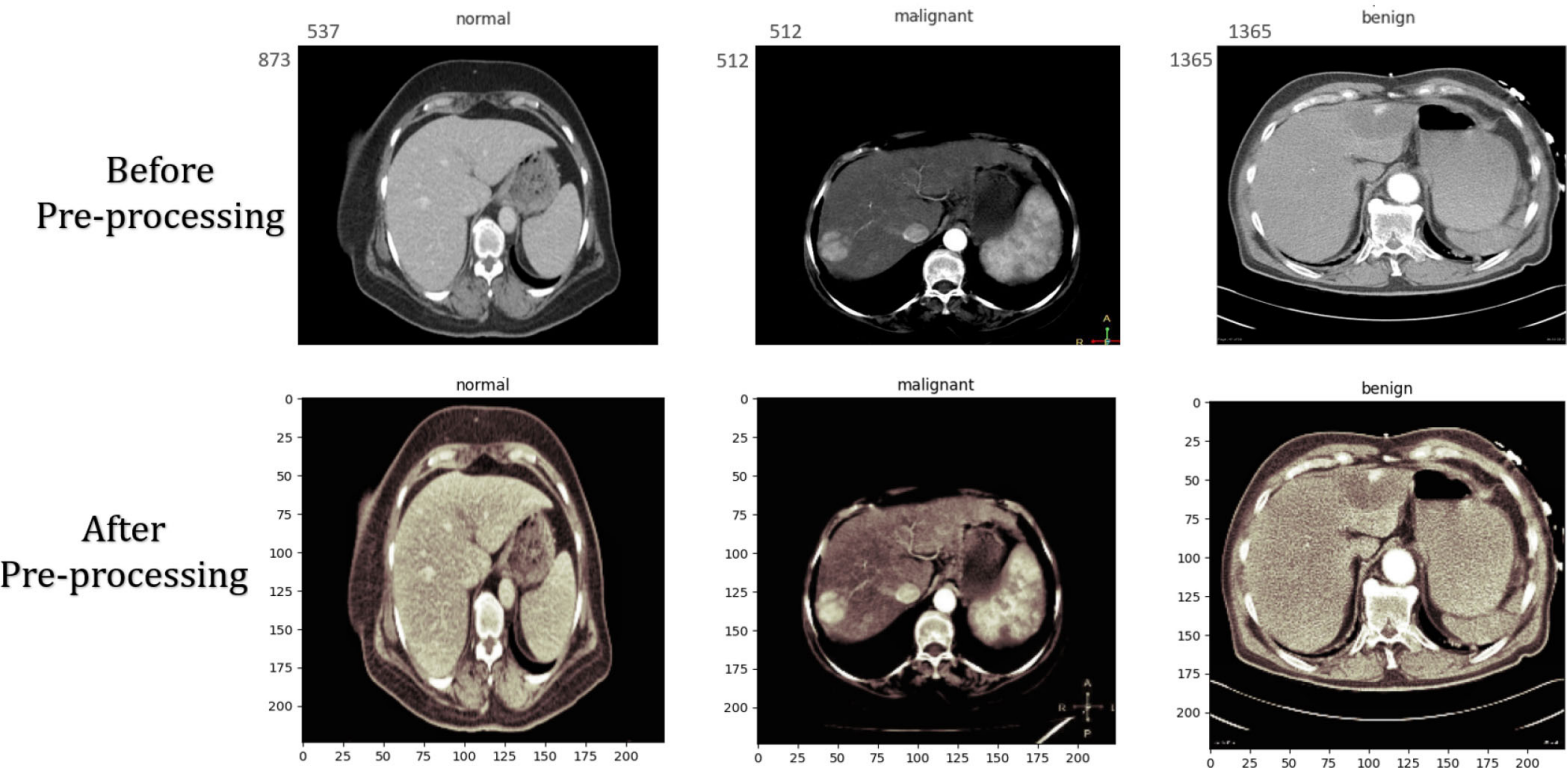
Preprocessing results for CT liver tumor images. Example CT scans before and after applying preprocessing (bilateral filtering and histogram equalization), demonstrating noise reduction and improved contrast.

As for the pre-processing process, five steps were performed as follows:

- Step (1): We used cv2 and os library to read the data and convert the images to grayscale.
- Step (2): The bilateral filter was applied to the images to reduce noise while preserving edges.
- Step (3): To improve the image’s contrast, the CLAHE (Contrast Limited Adaptive Histogram Equalization) algorithm was applied.
- Step (4): We utilized a pseudocolor map that enhances the contrast of grayscale images while visualizing them. It allocates distinct colors to varying intensity values, creating a visually appealing representation.
- Step (5): Due to the different sizes of the collected images, the image must be resized, which was necessary to standardize the input size for further processing or model requirements, and it has been performed in this step, **Figure 3** shows the images before and after pre-processing.

Four neural network architectures have been experimented with to assess the classification’s performance of our system. The classification model utilizes a folder structure to define the class names. Consequently, each image in the dataset is assigned a class according to its stored folder. The CT images have different pixel sizes, so we apply a resizing process to (224 X 224). During training, the learning rate (lr) starts at 0.001 and decreases if the validation loss stops improving using the ReduceLROnPlateau callback function from the Keras framework. However, this function was employed explicitly for the CNN models. In the case of ViT 16, the learning rate was reduced by a factor of 0.1 at epochs 30, as seen in Dosovitskiy et al. (34). The step learning rate schedule factor is a frequently employed technique to progressively decrease the learning rate throughout the training process, enabling the model to achieve more efficient convergence. Moreover, the learning rate is dynamically modified for each parameter according to the magnitude of the gradients because we employed the Adam (Adaptive Moment Estimation) algorithm as the optimization algorithm. For batch size, after several tests, we determined that a batch size of 20 was optimal for our resource capabilities and helped to produce robust results. It was empirically determined that choosing an Epoch count of 50 guarantees efficient training, as this is the point around which most of the training converges. It is worth noting that all these settings have been performed for all four algorithms. For the loss function, ‘categorical cross-entropy’ has been selected because we have a multi-class dataset. In addition, early stopping was applied based on the validation loss, training is stopped if the validation loss did not improve for 10 epochs, and the best-performing weights were stored, that helps to prevent model overfitting; the **Table 4** summarizes all these setup procedures mentioned above.

**Table 4:** Summary of the classification models hyperparameter setting.

Hyperparameter	Value
Input image size	224 × 224 × 3
Base learning rate	1e-03
Batch size	20
Optimizer	Adam

### 4.2 Evaluation metrics

A comprehensive set of metrics is employed to assess the optimal and comparable baseline estimates similar to the previous studies. The evaluation tools used for the classification and explainable models are presented in this subsection. There are many measurement tools to evaluate the quality of classification, including Accuracy, Precision, Recall, and F1 score, as follows:

• Accuracy (Equation 8): One of the essential aspects to consider in the context of test samples. It refers to the proportion of accurate forecasts, encompassing correct positive and negative predictions, divided by the overall number of predictions made. The formula is defined as follows:

(8) $$\text{Accuracy}=\frac{TP+TN}{TP+TN+FN+FP}$$

Where TP is true positive, TN is true negative, FP is false positive, and FN is false negative.

• Precision (Equation 9): also known as the positive predictive value (PPV), is not frequently employed to validate medical images. However, it is utilized to compute the F1- Score Taha and Hanbury (54). The following formula defines this term:

(9) $$Precision=\frac{TP}{TP+FP}$$

• Recall (Equation 10): True Positive Rate (TPR), often referred to as Sensitivity, is a metric used to evaluate the performance of a classification model. It measures the ability of the model to identify positive instances and discard false positive predictions correctly; the formula is defined as follows:

(10) $$Recall=Sensitivity=\frac{TP}{TP+FN}$$

• F1 score (Equation 11): Classification is a machine learning activity widely associated with this metric; it represents the harmonic average value of the precision and recall estimates as the following formula illustrates:

(11) $$F1-score=2*\frac{Precision*Recall}{Precision+Recall}$$

• The Area Under the ROC Curve (AUC): It is an accuracy assessment in diagnostic radiology commonly performed using the area under the receiver operating characteristic (ROC) curve (AUC) Taha and Hanbury (54). The ROC curve is the plot of TPR against FPR, and AUC is the case where a test segmentation is compared to a ground truth segmentation. A larger AUC value indicates the superior performance of the model in effectively discerning between positive and negative classes Taha and Hanbury (54).

## 5 Experimentation results

In this section, we present the research’s implementation procedure and discuss the results and findings made from executing this work on several deep learning algorithms for the classification approach. The following sections discuss the results.

### 5.1 Tumor classification performance

The Workflow of the Classification Method in the previous chapter (**Figure 2**) demonstrates that several processes must be executed before obtaining the results. The necessary libraries were initially installed, followed by the implementation of data pre-processing. Subsequently, various deep learning algorithms were employed to train and assess performance. The results from each architecture used in the classification approach are shown in detail.

**Figure 4:**
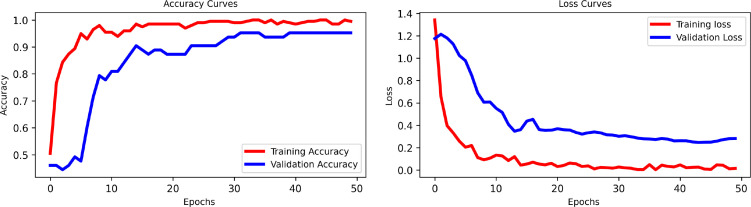
ResNet50-V2 training curves. Accuracy and loss progression during training and validation, highlighting model stability and convergence.

**Figure 5:**
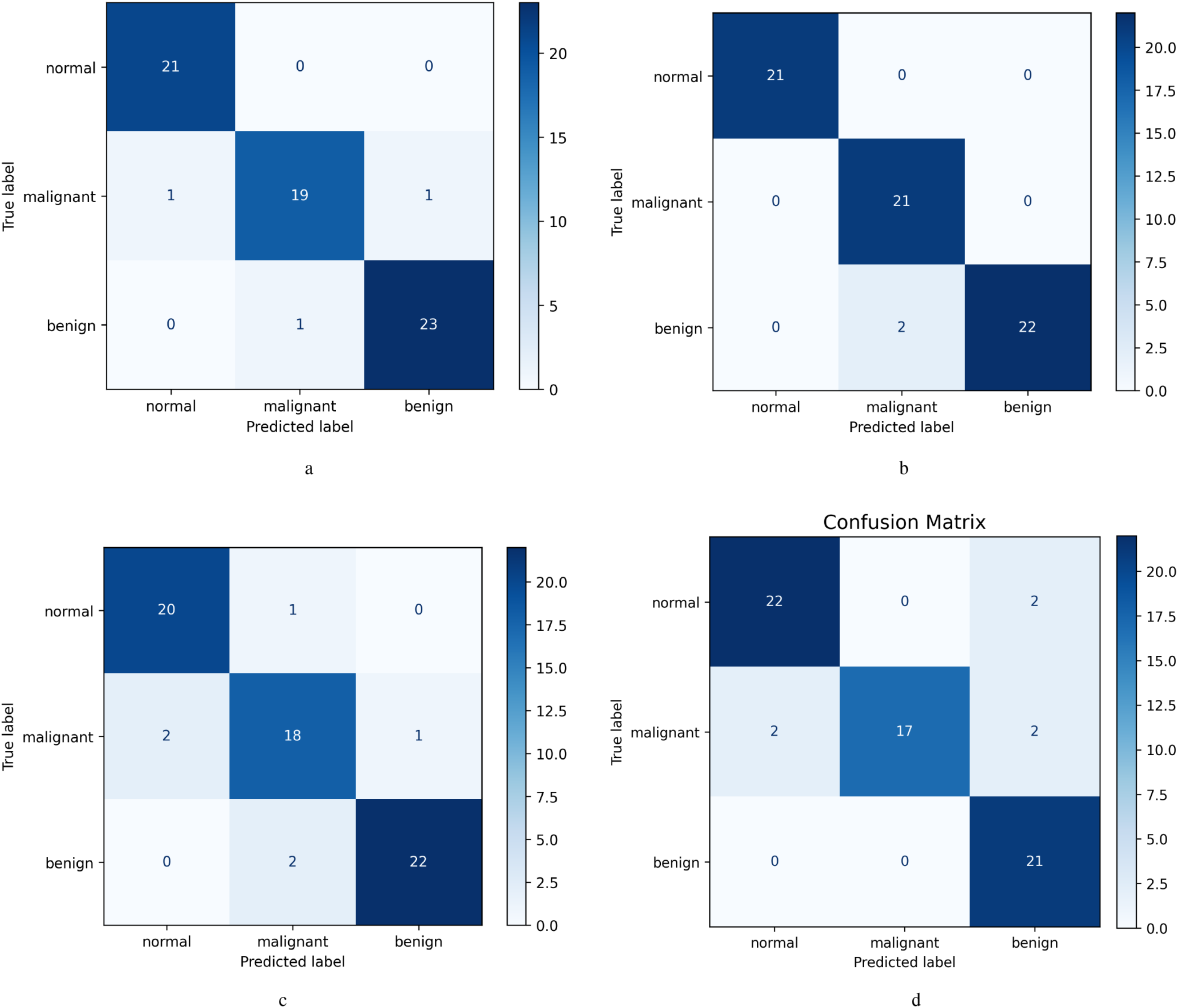
Confusion matrices for tumor classification models. Comparison of classification performance across **(a)** ResNet50-V2, **(b)** EfficientNetV2-S, **(c)** Inception-V3, and **(d)** ViT-16.

**Figure 6:**
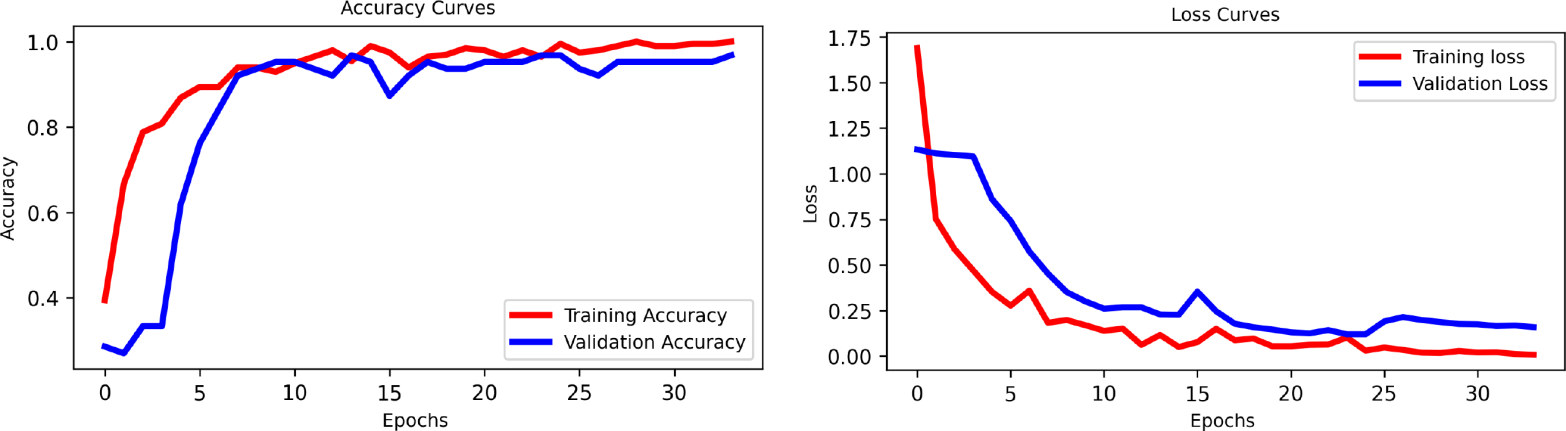
EfficientNetV2-S training curves. Accuracy and loss curves for EfficientNetV2-S, showing its performance across epochs.

**Figure 7:**
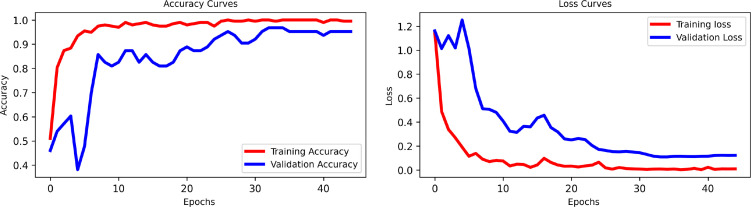
Inception-V3 training curves. Accuracy and loss behavior for Inception-V3, reflecting multiscale feature learning efficiency.

**Figure 8:**
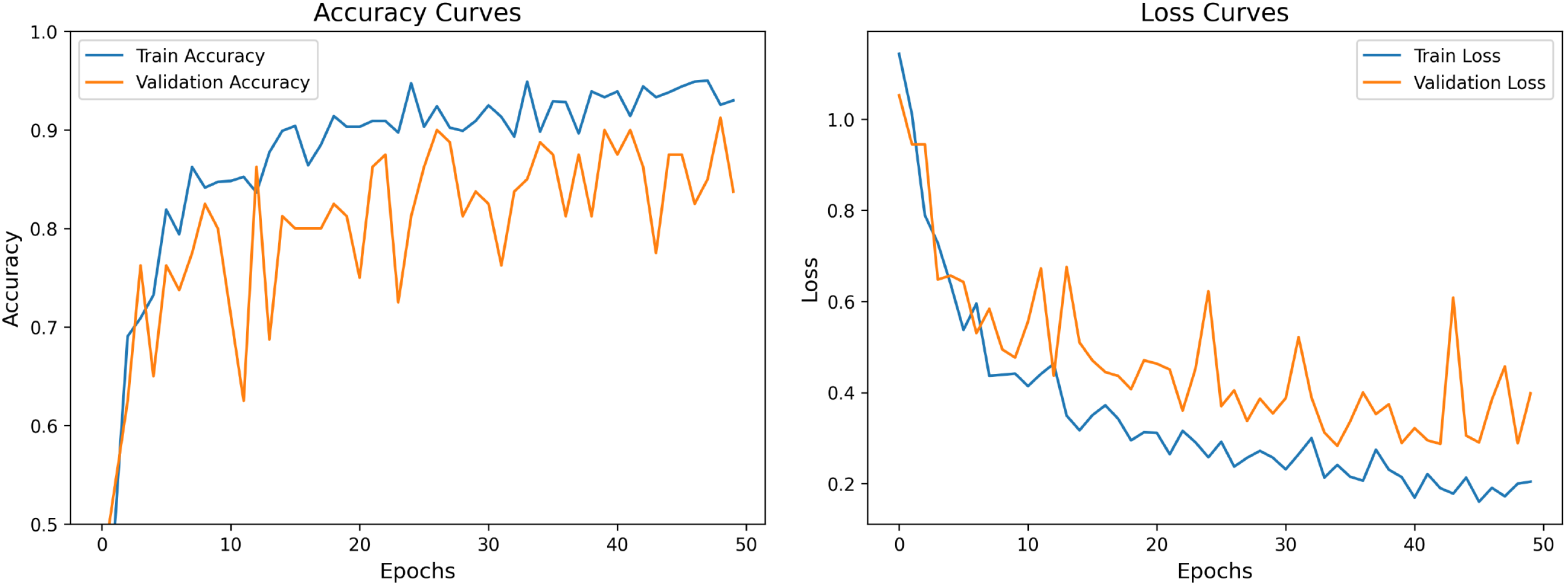
ViT-16 training curves. Accuracy and loss curves for Vision Transformer (ViT-16), illustrating its data dependency and training behavior.

- Residual Network (ResNet50-V2): Several experimentations have been conducted to evaluate the ResNet50-V2 model. ResNet50-V2 results vary between 100-97% for the training, 100-94% for validation, and 96-89% for the testing set. We have yet to find a similar work focusing on the classification of CT images for liver tumors using ResNet50-V2, as we have addressed in this work, so we can compare the performance of this model with it. However, despite the small data in this research, the results of this model in some experiments were better than those of the two studies that used ResNet50-v2 with tumor classification for other organs Chou et al. (55)Rahimzadeh and Attar (56) since their accuracies ranged between 89-91%. **Figure 4** shows the most frequent results we obtained from the ResNet50-V2 classifier. It illustrates the accuracy and loss curves for the training and validation set. The graph reveals that after epoch 31, the accuracy of the validation set and the loss saturated, and there was no change, so it stopped iterating. Whereas **Table 5** demonstrates the values for accuracy and loss for the training, validation, and testing set. **Figure 5a** shows the confusion matrix for the ResNet50-V2 model. There is one FN for the class malignant, which becomes an FP for the class normal. Also, one FN for the class malignant becomes an FP for the class benign. As for benign class, one FN becomes an FP for the class malignant. All the images of the class normal have been predicted correctly.
- EfficientNetV2: The EfficientNetV2-S architecture was also selected since it offers a compromise between training speed, model size, and computational efficiency. This is achieved by utilizing various combinations of MBConv and fused-MBConv blocks. It achieves the best and most stable results among the used architectures. The model was trained multiple times after setting the hyperparameters, as **Table 4** shows. **Figure 6** shows the model’s accuracy and loss at different training steps. The model achieved 96% accuracy for training data; for the validation set, the best accuracy was 96%, and the training was completed in 7m 46s. However, when evaluating the model after reloading it again, we obtained 96% accuracy for both validation and testing; **Table 6** shows the score achieved by the model. **Figure 5b** displays the EfficientNetV2 confusion matrix; there are two FNs for the class benign, which become an FPs for the class malignant. On the other hand, all the images of normal and malignant classes have been predicted correctly.
- Inception-V3: Although Inception-V3 seems to have a complicated architecture, it achieved more stable results than ResNet50-V2. From **Figure 7**, we can notice that the curve of the Inception-V3 model has less fluctuation than the ResNet50-V2 curve. When observing **Table 7**, even though the accuracy of the testing set in Inception-V3 is less than ResNet50-V2, as shown in **Table 5**, the loss for the same set was less than ResNet50-V2, which indicates the instability of the latter model. The confusion matrix of the Inception-V3 model in **Figure 5c** shows false negative samples for each class. For the class normal, there is one FN (False Negative), which becomes an FP (False Positive) for the class malignant. Also, two FNs for the class malignant become FPs for the class normal, and one FN in the malignant class becomes an FP for the class benign. For the benign class, two FNs become FPs for the malignant class.
- Vision Transformer ViT-16: We added ViT 16 as one of the architectures in our classification approach since it was mentioned in Dosovitskiy et al. (34) that by training the ViT 16 model on extensive data and applying it to various image recognition benchmarks such as ImageNet, CIFAR-100, and VTAB, the Vision Transformer (ViT) achieves outstanding performance compared to advanced convolutional networks. Moreover, the ViT model requires significantly fewer computational resources for training. However, when the models were trained on datasets of medium size, their accuracy was relatively low compared to architectures similar to ResNet. However, looking at **Figure 8**, which shows the accuracy and loss curves for training and validation, and regarding their observed struggle to reach the global minima, the gap between the training and validation is much less than ResNet50-V2 and Inception-V3; as for the accuracy, ViT 16 achieved similar results to Inception-V3 regarding testing accuracy. **Table 8** demonstrates a clearer view of this progress in performance. **Figure 5d** exhibits the ViT 16 confusion matrix; there are two FNs for the normal class, which become FPs for the benign class. Also, two FNs for the malignant class become FPs for the normal class, and two FNs for the malignant class become FPs for the benign class. For the benign class, all the images have been predicted correctly.

**Table 5:** ResNet50-V2 accuracy and loss values.

Metric	Training	Validation	Testing
Accuracy [%]	99.49	95.24	95.45
Loss	0.0260	0.2470	0.1593

**Table 6:** EfficientNetV2 accuracy and loss values.

Metric	Training	Validation	Testing
Accuracy [%]	96.46	96.83	96.97
Loss	0.1021	0.1195	0.1253

**Table 7:** Inception-V3 accuracy and loss values.

Metric	Training	Validation	Testing
Accuracy [%]	100	96.83	90.91
Loss	0.0053	0.1088	0.3203

**Table 8:** ViT-16 accuracy and loss values.

Metric	Training	Validation	Testing
Accuracy [%]	93.00	83.75	90.01
Loss	0.2043	0.3983	0.2568

As observed in **Figure 5**, the models demonstrated high overall performance, some common misclassifications were observed. For instance, almost all models misclassified malignant cases as benign and vice versa; this occurs because the similarity and overlap in their visual characteristics. Moreover, few malignant cases were classified as normal. These failures most likely occurred due to the limited size and heterogeneous appearance of malignant tumors, which can reduce the models’ ability to fully generalize across all cases.

### 5.2 Discussion

We compared the performance of our models with each other. **Table 9** shows this comparison using five distinct measures employed to assess the quality of their image data classification. From **Table 9**, EfficientNetV2-S has the highest results in accuracy and for other metrics it is similar to ResNet50-V2, where ResNet50-V2 has the highest result in AUC. For ViT 16 and Inception-V3, we observe a similarity with slightly higher F1-Score and Precision for Inception-V3, while Inception-V3 has higher AUC than ViT-16. Also, to validate the results, 95% Confidence Intervals (CIs) were applied using bootstrapping with 2,000 iterations. **Table 10** shows the same metrics using K-Fold cross-validation, and EfficientNetV2-S achieved the highest results in all metrics. Finally, **Table 11** shows the total number of parameters for each model, number of mathematical operations (FLOPs) produced using keras flops library. ViT-16 had the shortest training time, with 50 total epochs. To contextualize the deep learning gains, we trained a Logistic Regression baseline on handcrafted features (intensity statistics, histogram, GLCM textures). The baseline reached 83.33% accuracy and 0.9513 AUC (**Table 9**), 13.6 points below EfficientNetV2-S.

**Table 9:** Evaluation metrics for the four models and the baseline [point estimates with 95% confidence intervals (CIs)].

Model	Accuracy [%]	F1-Score [%]	Recall [%]	Precision [%]	AUC
ResNet50-V2	95.45 (89.4–100.0)	95.42 (89.3–100.0)	95.45 (89.4–100.0)	95.45 (90.1-100.0)	0.9958 (0.987-1.0)
EfficientNetV2-S	96.97 (92.4–100.0)	95.42 (92.4–100.0)	95.45 (92.4–100.0)	95.45 (93.9–100.0)	0.9948 (0.984–1.0)
Inception-V3	90.91 (83.3–97.0)	90.01 (83.3–97.0)	90.91 (83.3–97.0)	90.98 (84.0–97.2)	0.9828 (0.958–0.997)
ViT-16	90.01 (83.3–97.0)	90.85 (83.4–97.0)	90.91 (83.3–97.0)	91.88 (86.8–97.2)	0.9783 (0.947–0.999)
Logistic Regression (baseline)	83.33 (74.2–92.4)	83.16 (73.9–92.3)	83.33 (74.2–92.4)	83.81 (75.8–92.6)	0.9513 (0.899–0.989)

**Table 10:** The evaluation metrics for the four models using K-Fold cross-validation.

Model fold 1		Fold 2	Fold 3	Fold 4	Fold 5	Avg
ResNet50-V2						
Accuracy [%] 96.97		90.91	89.23	93.85	83.08	90.81
Precision [%] 96.97		91.56	89.67	93.92	86.21	91.67
Recall [%] 96.97		90.91	89.23	93.85	83.08	90.81
F1-score [%] 96.97		90.83	88.76	93.85	83.13	90.71
AUC 0.9956		0.9857	0.9774	0.9927	0.9399	0.9782
EfficientNetV2-S						
Accuracy [%] 93.94		96.97	95.38	96.92	87.69	94.18
Precision [%] 94.20		97.23	95.37	96.99	91.09	94.98
Recall [%] 93.94		96.97	95.38	96.92	87.69	94.18
F1-score [%] 93.95		96.97	95.34	96.92	87.80	94.20
AUC 0.9979		0.9996	0.9925	0.9996	0.9949	0.9969
Inception-V3						
Accuracy [%]	92.42	89.39	92.31	93.85	75.38	88.67
Precision [%]	93.88	92.05	92.40	94.25	78.09	90.13
Recall [%]	92.42	89.39	92.31	93.85	75.38	88.67
F1-score [%]	92.39	89.48	92.22	93.82	73.72	88.33
AUC	1.0000	0.9901	0.9754	0.9971	0.9617	0.9848
ViT-16						
Accuracy [%]	90.91	87.88	87.88	89.23	90.77	89.33
Precision [%]	91.45	87.98	88.74	91.92	92.31	90.48
Recall [%]	90.91	87.88	87.88	89.23	90.77	89.33
F1-score [%]	90.80	87.71	88.09	89.47	90.68	89.35
AUC	0.9767	0.9712	0.9560	0.9661	0.9918	0.9724

**Table 11:** Model complexity and training summary for each architecture. Parameters are reported in millions (M).

Model	Parameters (M)			FLOPs	Training time	Epochs
	Total	Trainable	Non-trainable	(GFLOPs)		
ResNet50-V2	76.92	25.62	0.05	6.99	6m 22s	44 (early stop)
EfficientNetV2-S	60.71	20.18	0.16	5.75	7m 46s	24 (early stop)
Inception-V3	71.65	23.87	0.04	5.70	6m 33s	35 (early stop)
ViT-16	85.80	0.004	85.80	11.29	6m 53s	50 (no early stop)

To further validate our results for the four models, an additional statistical analyses were applied. **Table 12** shows pairwise statistical comparison of models using McNemar’s test (accuracy) and DeLong’s test (AUC, One-vs-Rest). McNemar’s test on classification accuracy shows that EfficientNetV2-S model demonstrated higher accuracy than ViT-16 (*p* = 0.00031), while ResNet50-V2 outperformed both Inception-V3 (*p* = 0.01562) and ViT-16 (*p* = 0.00002). On the other hand, DeLong’s test showed that ViT-16 has different AUC values than ResNet50-V2 on the normal (*p* = 0.01198) and benign (*p* = 0.00973) classes, as well when comparing it with EfficientNetV2-S and Inception-V3 on the benign class (*p<* 0.0001). There was no significant difference in AUC between EfficientNetV2-S and ResNet50-V2 across any class.

**Table 12:** Reported values are exact *p*-values from McNemar’s test (Accuracy) and DeLong’s test (AUC, one-vs-rest per class).

Comparison	Mcnemar (Accuracy)	Delong (Normal)	Delong (Malignant)	Delong (Benign)
ResNet50-V2 vs EfficientNetV2-S	0.07835	0.58349	0.08391	0.07845
ResNet50-V2 vs Inception-V3	**0.01562**	0.13492	0.27332	0.13336
ResNet50-V2 vs ViT-16	**0.00002**	**0.01198**	0.32156	**0.00973**
EfficientNetV2-S vs Inception-V3	0.81453	0.50069	0.18968	0.23223
EfficientNetV2-S vs ViT-16	**0.00031**	**0.00000**	0.44018	**0.00000**
Inception-V3 vs ViT-16	**0.00072**	0.09421	0.06556	**0.00000**

Significant results (*p<* 0.05) are highlighted in bold.

An in-depth analysis of the results highlights several factors underlying the discrepancies among models. EfficientNetV2-S consistently outperformed other architectures owing to its compound scaling strategy, which optimally balances network depth, width, and resolution, thereby capturing intricate liver tumor features while mitigating overfitting. ResNet50-V2 achieved high accuracy and AUC, likely due to its residual connections that alleviate vanishing gradients and support stable training. Inception-V3, despite its ability to capture multi-scale features, struggled with small lesion boundaries, explaining its reduced recall and F1 scores. ViT-16 performed less favorably, reflecting its greater data requirements and vulnerability to limited training samples, although it has the theoretical advantage of modeling long-range dependencies. Overall, these findings suggest that convolutional inductive biases remain advantageous for medical imaging tasks with modest dataset sizes, whereas transformer-based models may require larger datasets to fully demonstrate their potential.

#### 5.2.1 Pseudocolor ablation

To validate the impact of the pseudocolor mapping step in our preprocessing pipeline, we applied an ablation study by retraining EfficientNetV2-S model on grayscale inputs (with grayscale stacked into 3 channels for compatibility with the pretrained backbone) and keeping all other hyperparameters the same. **Table 13** compares results before and after removing pseudocolor: accuracy reduced from 96.97% to 93.94%, F1-score from 95.42% to 93.97%, and AUC from 0.9948 to 0.9933. This confirms that pseudocolor contributed positively to classification performance without introducing artifacts.

**Table 13:** Ablation study on the pseudocolor preprocessing step (EfficientNetV2-S).

Configuration	Accuracy [%]	Precision [%]	Recall [%]	F1-score [%]	AUC
With pseudocolor (proposed)	96.97	95.45	95.45	95.42	0.9948
Without pseudocolor	93.94	94.43	93.94	93.97	0.9933

#### 5.2.2 Limitations

A primary constraint of our work is the limited access to extensive, publicly annotated three-class datasets of liver tumors, since most public CT datasets (e.g., LiTS, CHAOS) were released primarily for segmentation tasks rather than classification. The dataset used in this work was limited and focused on a specific hospital, which impacted the generalizability of the findings. The original providers also did not publish detailed annotation protocols, scanner metadata, or inter-observer agreement metrics, which we acknowledge as a limitation of the source datasets. The wide 95% confidence intervals reported in **Table 9** reflect this limited test set size and indicate that point estimates should be interpreted with caution. Despite utilizing data augmentation, cross-validation, and statistical analysis to address this issue, external validation on multi-institutional datasets is essential to verify robustness.

### 5.3 Interpreting model decisions

The need for model explainability has become indispensable and cannot be disregarded. With models’ rising complexity and power, it is crucial to comprehend and interpret their decisions. Model explainability facilitates establishing trust and credibility in the predictions and suggestions generated by deep learning models. When a model produces an output, it is essential not to accept it uncritically. We applied three different explanation models to the classification model that demonstrated the best performance in our work (EfficientNetV2-S).

**Figure 9:**
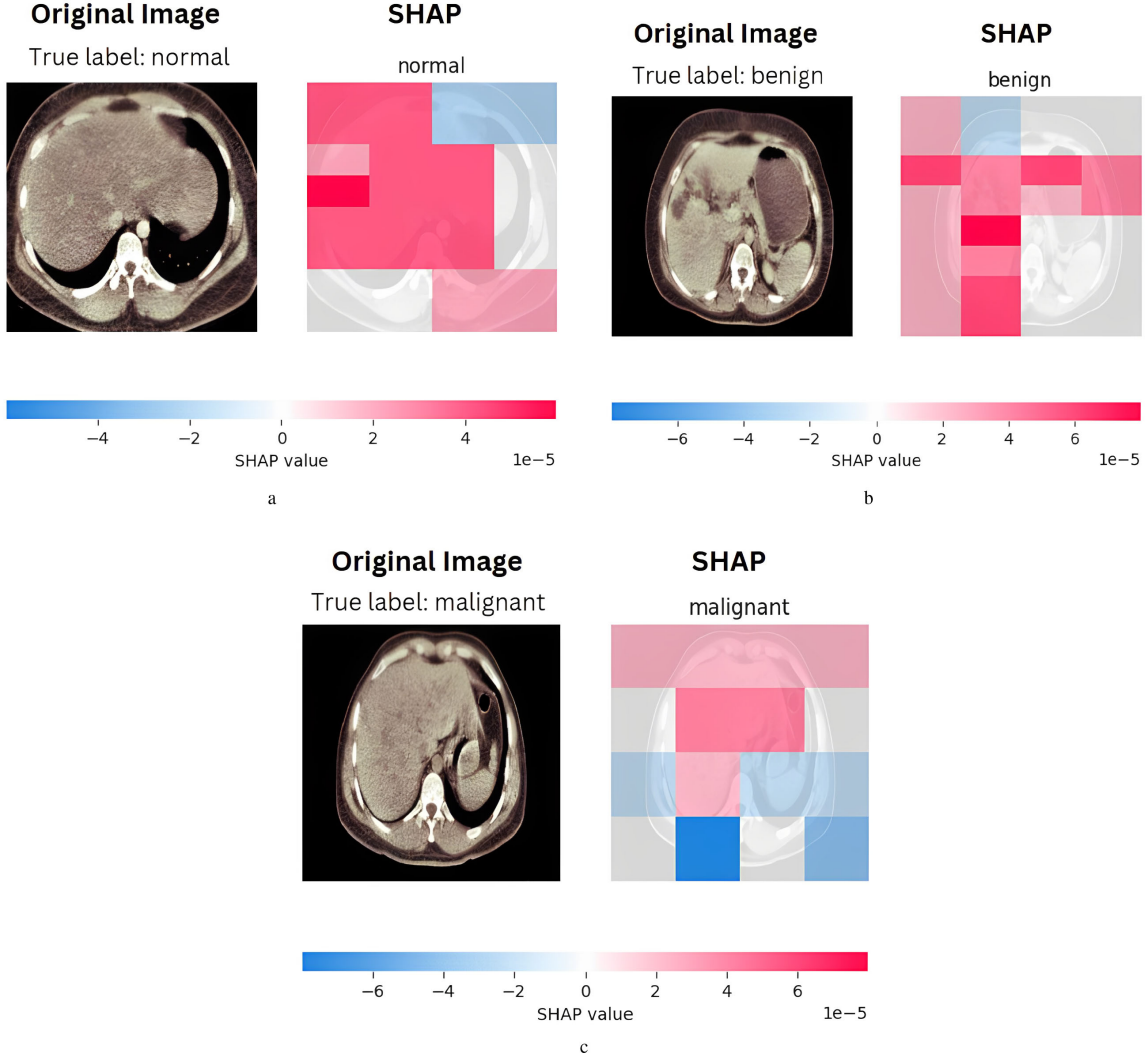
SHAP visualizations for tumor classification models. SHAP visualizations for **(a)** Normal, **(b)** Benign, and **(c)** Malignant cases, identifying influential image regions for predictions.

**Figure 10:**
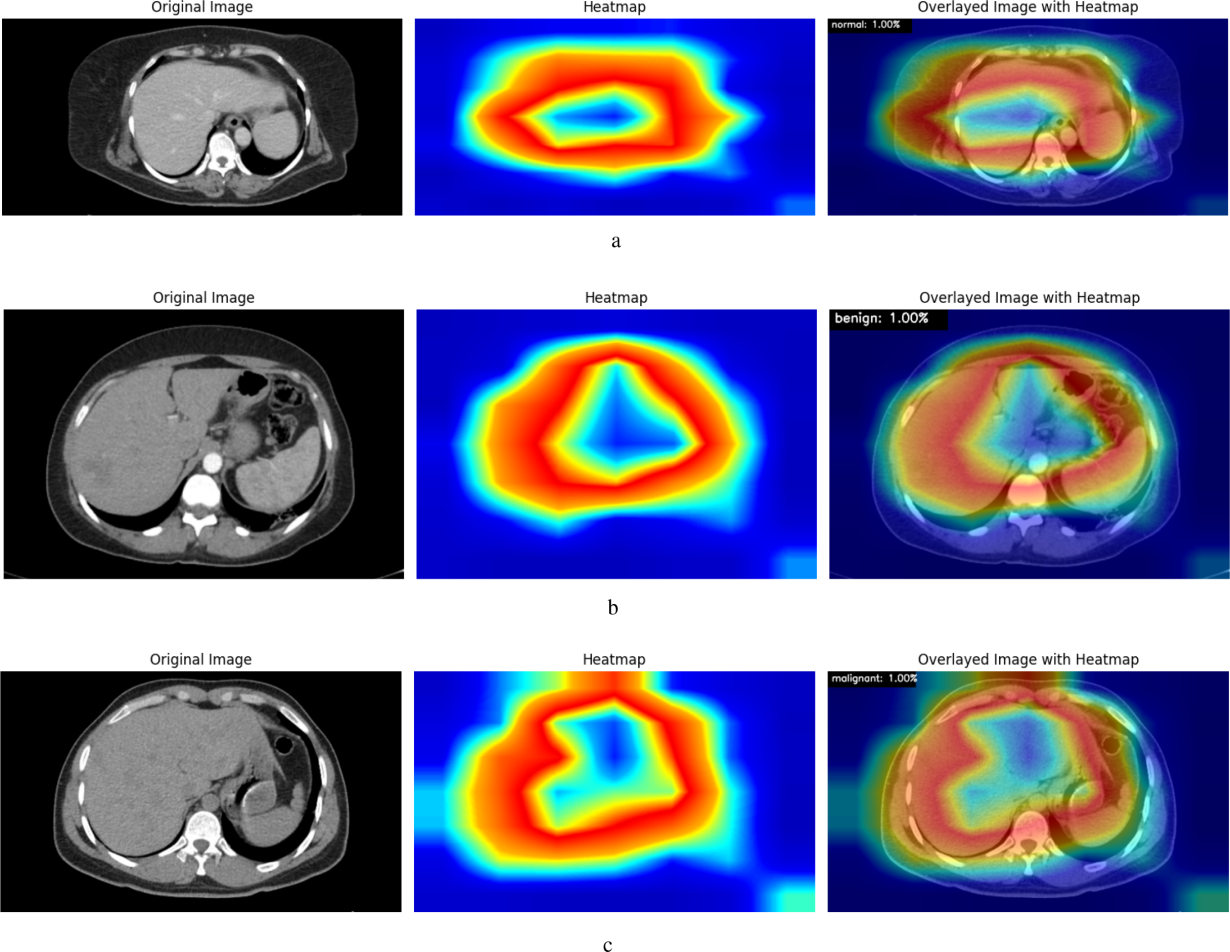
Grad-CAM visualizations of CT liver tumor predictions. Class activation maps for **(a)** Normal, **(b)** Benign, and **(c)** Malignant classes, highlighting discriminative image regions.

**Figure 11:**
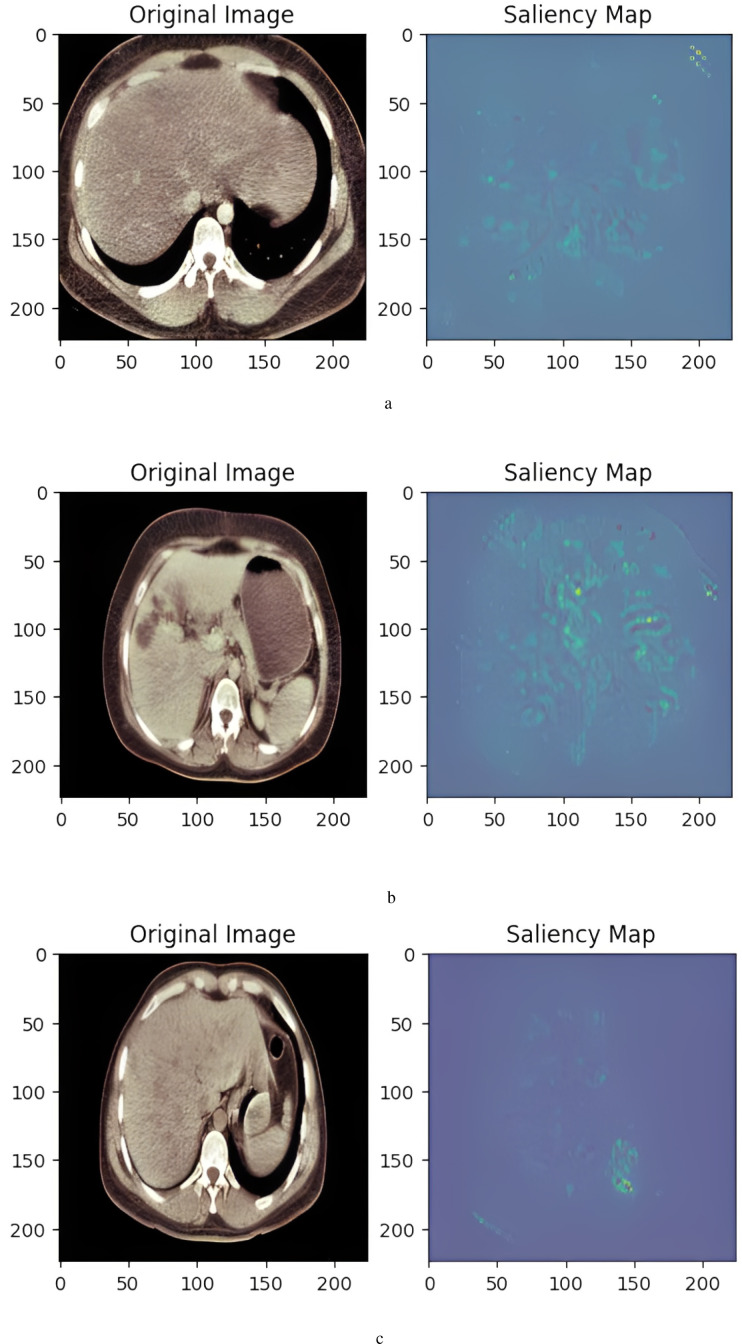
Saliency map interpretations of CT liver tumor images. Pixel-wise sensitivity maps for **(a)** Normal, **(b)** Benign, and **(c)** Malignant classes, emphasizing regions critical to model decisions.

- SHAP: SHAP aims to explain model predictions by quantifying the contribution of each feature to the forecast. The term refers to the mean incremental impact of a specific feature value over all potential alliances, so we are addressing local and global levels. In **Figure 9**, three original images belonging to three classes on the left side of the figure and the resulting images after applying SHAP on the right side. The images on the right side correspond to the SHAP values for the images on the left side; it is the original image with a colored mask, and the colors of that mask vary from blue to pink. Observing these colors allows for an assessment of the features under the mask on the prediction. “Blue” means this feature does not significantly impact the prediction, while “Pink” dramatically impacts the prediction. This process proves how much each attribute contributes to the ultimate forecast. In all three images, the prediction was correct.
- GradCAM: This method uses a specific layer, the last convolutional layer, then saves the output of this layer and the gradients from this layer not to update weights but to find the most affected area in the image to the prediction and then visualize it as a heatmap. **Figure 10** shows the GradCAM of the EfficientNetV2-S classifier; in each category, we have three images: original image, heatmap, and overlay image with heatmap. At the top of the “overly image with heatmap” image for all classes, the probability of this class is observed. Moreover, high highlights for some critical regions are observed, as expected. However, the image of class normal, **Figure 10h**, highlights some top and bottom regions of the liver, which is essential, as for the images of the benign and malignant classes, **Figures 10i, j**, the most critical areas were selected. At the same time, this method did not contribute any importance to the background, which was the case of the previous methods.
- Saliency Map: The saliency map could shows the importance of individual pixels within the input image and the overall predictive capabilities of CNN models. Each pixel in the saliency map is assigned a color or magnitude representing its importance in the classification process. Saliency maps are gradient-based visualization techniques that emphasize the most prominent areas of an input image that have the most impact on the model’s prediction; this is determined by analyzing the gradient of the model’s output for the input image pixels. From **Figure 11**, there is similarity on the areas that have the most impact on the prediction with Grad-CAM method. A close examination of the images that have the caption “Saliency map” in **Figure 11**, we can observe the following points:
- For the image of the class normal, **Figure 11k** shows that many pixels represent the liver. However, some unrelated pixels are highlighted at the top-right side of the image.
- In the image of the benign class, **Figure 11l**, many pixels representing the top-center of the liver and the right side of the image have been colored differently, which means they are essential in the classification process.
- As for **Figure 11m**, which shows the image from the malignant class, we observe some pixels at the top-center of the liver, while the pixels at the bottom of the image contribute the most to the prediction decision.

Our results demonstrate that EfficientNetV2-S consistently attained superior performance in terms of accuracy, F1-score, recall, and AUC, exhibiting strong outcomes under both bootstrapping and k-fold cross-validation. In contrast to previous studies, including Meng et al. Meng et al. (10), which focused on hybrid feature extraction for segmentation, and Balasubramanian et al. Balasubramanian et al. (7), which utilized a Swin Transformer for classification, our approach attains similar or superior accuracy while distinctly including explainable AI. This combination directly resolves the interpretability gap identified in previous studies, hence enhancing confidence in therapeutic environments.

The ramifications of this work encompass both research and practice. Integrating explainable deep learning models into CT-based liver tumor classification may diminish reliance on invasive biopsies, assist radiologists in intricate cases, and enhance patient outcomes. Future research priorities involve verifying the framework using multi-institutional datasets, integrating multi-modality imaging, and providing a publicly accessible dataset to enhance reproducibility and collaboration in liver tumor classification research.

## 6 Conclusions

This work introduced a comprehensive deep learning framework for the non-invasive categorization of liver cancers using CT scans, overcoming the constraints of conventional biopsy and manual radiological analysis. Through a rigorous assessment of various advanced architectures—ResNet50-v2, EfficientNetV2, Inceptionv3, and ViT-16—alongside customized preprocessing and augmentation techniques, the framework attained commendable performance, with EfficientNetV2 achieving an accuracy of 96.97%. The use of explainable AI (XAI) methodologies guarantee transparent decision-making, reconciling the disparity between high performing algorithms and therapeutic trust.

In contrast to previous studies that emphasized handcrafted features, utilized limited datasets, or lacked interpretability, our methodology provides a scalable, interpretable, and clinically relevant solution for liver tumor classification. This progress enables the development of dependable AI-assisted diagnostic tools that enhance the accuracy and efficiency of radiologists.

Notwithstanding these encouraging outcomes, other problems persist. A significant constraint is the limited access to extensive, publicly annotated datasets, which impedes reproducibility and generalizability. Future endeavors will concentrate on partnering with medical institutions to develop diverse, multi-institutional datasets that are publicly available. Additionally, we intend to expand this framework to encompass 3D CT volumes and incorporate automated segmentation of both liver and tumor regions, facilitating a comprehensive end-to-end pipeline for diagnosis, treatment planning, and surgical decision making. 

## Data Availability

The original contributions presented in the study are included in the article/supplementary material. Further inquiries can be directed to the corresponding author.
